# Comparative effectiveness of nerve block strategies for preventing postherpetic neuralgia in thoracic herpes zoster: a network meta-analysis

**DOI:** 10.3389/fneur.2025.1612871

**Published:** 2025-08-18

**Authors:** Wensheng Lu, Shengze He, Qi Liu, Yaozu Gu, Jie Bai

**Affiliations:** ^1^Department of Anesthesiology, Lanzhou University Second Hospital, Lanzhou, China; ^2^The Second Clinical Medical School, Lanzhou University, Lanzhou, China

**Keywords:** herpes zoster, nerve block, acute zoster-related pain, postherpetic neuralgia, erector spinae plane block, paravertebral block, network meta-analysis

## Abstract

**Background:**

Shingles is a common global disease, with the chest region being one of its most frequently affected areas. Postherpetic neuralgia (PHN) is one of the most common and debilitating complications of this disease, characterized by high incidence, prolonged duration, and treatment resistance, severely affecting patients’ daily life and quality of life. Currently, research on the prevention of PHN remains limited. Nerve block, as a promising intervention, has been widely applied in clinical pain management. However, there is still no consensus on the efficacy and safety of different nerve block techniques for the prevention of chest-related PHN, warranting further systematic evaluation and comparison.

**Methods:**

A network meta-analysis was conducted using RevMan 5.4 and Stata 18.0, analyzing data from nine studies retrieved from four English-language databases: MEDLINE, Embase, Web of Science, and the Cochrane Central Register of Controlled Trials (CENTRAL).

**Result:**

This study included a total of 9 randomized controlled trials, involving 741 patients with chest herpes zoster and 8 different interventions. The results of the network meta-analysis indicated that the top three interventions in terms of total effective rate were: PVB: Amide local anesthetics + Methylene blue > PVB: Methylene blue > ESPB: Amide local anesthetics + Glucocorticoid. In terms of pain level, as assessed by the Visual Analogue Scale, the top three interventions were: PVB: Amide local anesthetics + Glucocorticoid > PVB: Amide local anesthetics > ESPB: Amide local anesthetics + Glucocorticoid.

**Conclusion:**

The results of this study indicate that PVB is superior to ESPB in both reducing VAS scores and improving the total effective rate. Among the interventions, PVB: Amide local anesthetics + Methylene blue demonstrated the best performance in terms of total effective rate, while PVB: Amide local anesthetics + Glucocorticoid showed the most significant effect in reducing VAS scores.

**Systematic review registration:**

https://www.crd.york.ac.uk/prospero/, identifier CRD42024604329.

## 1 Background

Herpes zoster is a painful rash caused by the reactivation of the varicella-zoster virus (VZV). Approximately one million cases of herpes zoster occur annually in the United States, and about 95% of adults have latent VZV infection, making them at risk for the disease (1). Clinically, herpes zoster manifests as a vesicular rash along the dermatomal distribution of the affected nerve, with the thoracic region being the most commonly affected, followed by the cranial nerve and lumbar regions (2). The localized symptoms can range from mild itching or tingling to severe pain, often accompanied by systemic symptoms such as headache, fever, and photophobia (3), significantly impacting the patient’s daily life and quality of life.

Although the majority of patients experience resolution of herpes zoster-associated pain over the course of the disease, a subset of individuals develops postherpetic neuralgia (PHN), characterized by the persistence of neuropathic pain after the resolution of the rash (4). Currently, various treatment options for PHN are available, including systemic or topical pharmacological therapies such as the application of 2% lidocaine gel four times daily (5), opioid analgesics (6), and anticonvulsants (7). However, research on the prevention of PHN remains limited, with only a few intervention strategies reported in the literature (8). Given that herpes zoster lesions in the thoracic region typically present as unilateral and localized, clinical practice has gradually incorporated interventional approaches, such as peripheral nerve blocks and axial nerve blocks, for the prevention and treatment of PHN (9–11). However, the efficacy and safety of various types of nerve block techniques—including epidural block, paravertebral block (PVB), erector spinae plane block (ESPB), intercostal nerve block, and stellate ganglion block—in preventing thoracic PHN remain inconclusive.

Therefore, this study aims to conduct a network meta-analysis to systematically compare the efficacy of different nerve block strategies in preventing postherpetic neuralgia in thoracic herpes zoster, with the goal of identifying the most optimal intervention strategy and providing evidence-based guidance for clinical practice.

## 2 Methods

### 2.1 Protocol and registration

This study adheres to the Preferred Reporting Items for Systematic Reviews and Meta-Analyses (PRISMA) guidelines for systematic reviews and meta-analyses (12), and has been registered with the International Prospective Register of Systematic Reviews (PROSPERO), with the registration number: CRD42024604329.

### 2.2 Search strategy

We conducted a systematic search of MEDLINE, Embase, Web of Science, and the Cochrane Central Register of Controlled Trials (CENTRAL), using a combination of subject headings and free-text terms. The search covered the period from the inception of each database to April 9, 2025. The detailed search strategy is provided in the Appendix 1. No language or publication type restrictions were applied during the search. All search results were imported into EndNote X9 reference management software, where duplicates were first removed automatically, followed by manual screening to eliminate any remaining duplicates. Additionally, we reviewed the reference lists of included studies to identify any potentially missed relevant articles.

### 2.3 Inclusion and exclusion criteria

Inclusion Criteria: (1) Study design: Only randomized controlled trials (RCTs) were included; (2) Study population: Patients diagnosed with following thoracic herpes zoster; (3) Intervention: The experimental group received different types of nerve block techniques, including but not limited to epidural block, paravertebral block, dorsal root ganglion block, intercostal nerve block, and stellate ganglion block. The control group received either a placebo or no intervention; (4) Outcome measures: At least one of the following outcomes was reported: the incidence of postherpetic neuralgia, pain intensity scores at different time points, and the time to onset of pain relief.

Exclusion Criteria: (1) Non-original research types, such as review articles, case reports, case series, editorials, commentaries, conference abstracts, basic experimental studies, or other studies unrelated to the topic; (2) Studies where other medications were combined with the intervention, introducing confounding factors; (3) Studies with incomplete data or unavailable full-text articles; (4) Duplicate publications or studies that were not peer-reviewed; (5) Non-english studies.

### 2.4 Data extraction

The literature screening process was conducted independently by two reviewers. Initially, duplicates were removed automatically using EndNote X9 software, followed by a preliminary screening of titles and abstracts to exclude studies that did not meet the inclusion criteria. For studies that potentially met the criteria after initial screening, the full text was reviewed to determine whether they satisfied the final inclusion and exclusion criteria. Data extraction was performed using a pre-designed standardized form, and the extracted information included: first author, year of publication, journal name, article title, country or region of the study, age range of participants, sample size, interventions, and outcome measures. In case of disagreements between the two reviewers during the screening or data extraction process, a third reviewer was consulted to resolve the issue through consensus.

### 2.5 Quality assessment

Two reviewers independently assessed the risk of bias in the included studies according to the evaluation criteria outlined in the Cochrane Handbook for Systematic Reviews of Interventions (version 5.1.0). The assessment tool included seven domains: random sequence generation, allocation concealment, blinding of participants and personnel, blinding of outcome assessment, incomplete outcome data, selective reporting, and other potential biases. Each domain was rated as “low risk,” “unclear risk,” or “high risk.” In cases of disagreement between the two reviewers, a third reviewer was involved in the discussion to make a final decision.

### 2.6 Statistical analysis

Network meta-analysis was performed using Stata software, and a network diagram was generated to compare the efficacy of different nerve block strategies in preventing postherpetic neuralgia in thoracic herpes zoster, while ranking the priority of each intervention. The network diagram was used to illustrate the comparative relationships between interventions, with the size of the nodes reflecting the sample size of each intervention included in the studies, and the lines representing direct comparisons between interventions. The thickness of the lines indicated the number of relevant studies. Before data synthesis, the assumptions for network meta-analysis, including transitivity and consistency, were tested to assess potential inconsistencies within the network structure. All analyses were conducted using random-effects models, and continuous variables were presented as mean differences (MD) with 95% confidence intervals (95% CI). Statistical significance was considered when the 95% CI of the MD did not include 0. The Surface Under the Cumulative Ranking Curve (SUCRA) was calculated to rank the interventions. The SUCRA values ranged from 0 to 100%, with higher values closer to 100% indicating better efficacy, and lower values closer to 0 suggesting poorer efficacy (13).

## 3 Results

### 3.1 Literature selection

**Figure 1:**
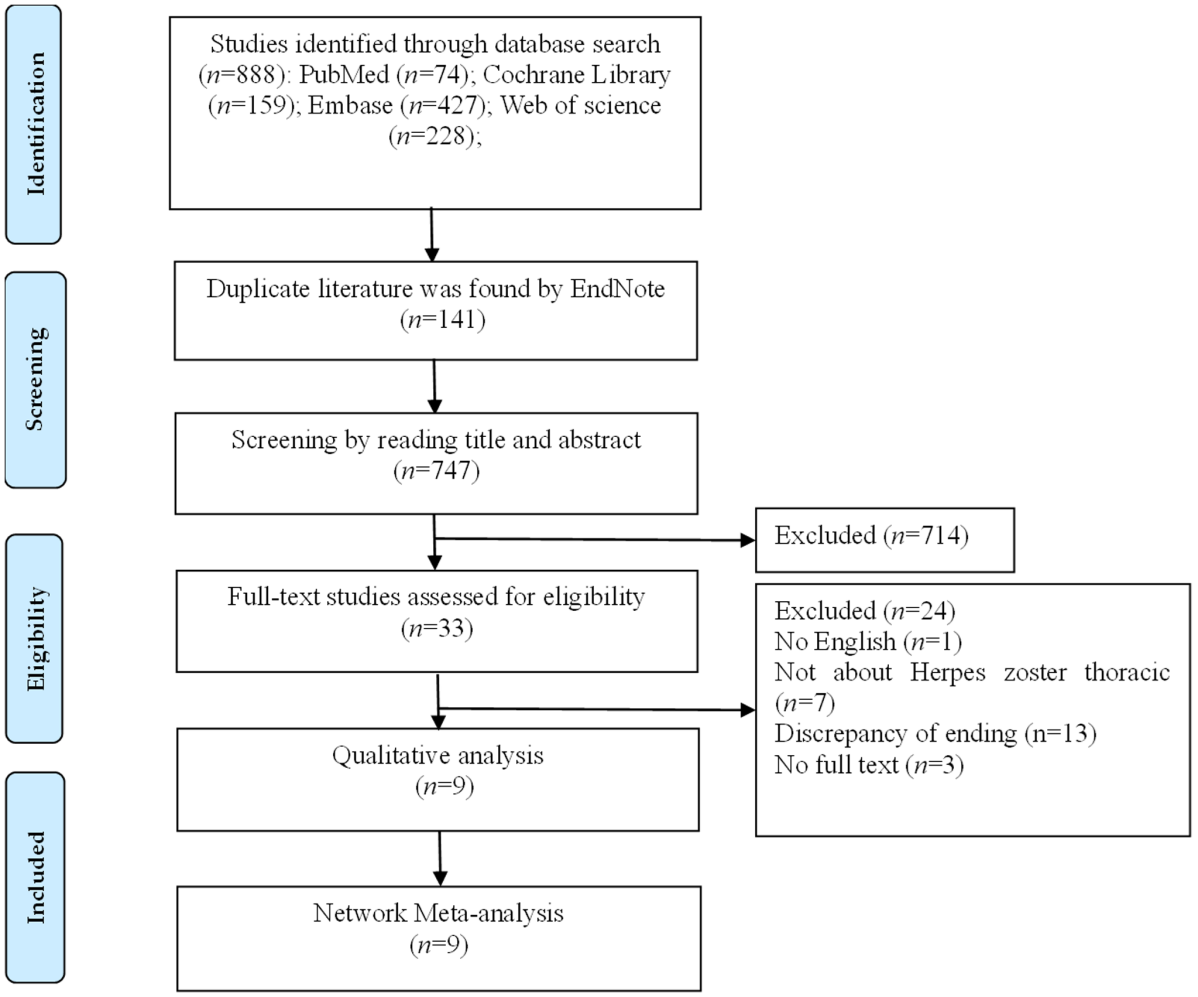
Document screening flow chart.

A total of 888 records were initially retrieved. After removing 141 duplicates, 747 records remained for title and abstract screening. Of these, 33 studies were selected for full-text review based on the inclusion criteria. Following detailed assessment, 9 randomized controlled trials were ultimately included in the network meta-analysis. A total of 741 patients with thoracic shingles and 8 interventions were involved. The literature selection process is illustrated in Figure 1.

### 3.2 Characteristics of included studies

The detailed characteristics of the included studies are summarized in Table 1.

**Table 1:** Features included in the study.

Author/Year	Country	Type of study	Sample size intervene/control	Age intervene/control	Intervention measure		Outcomes
					Intervene group	Control group	
Zhao and Mei (2019) (32)	China	RCT	43/44	57.4 ± 8.3/ 52.8 ± 7.3	PVB: Methylene blue	Antiviral therapy	①, ②
Ma et al. (2022) (33)	China	RCT	50/50	60,51 ± 10,69/ 63,93 ± 10,72	PVB: Amide local anesthetics	Antiviral therapy	①
Ji et al. (2022) (34)	China	RCT	36/36	71.55 ± 9.13/ 69.22 ± 6.97	PVB: Methylene blue	Placebo	①
El-Sayed et al. (2021) (35)	USA	RCT	20/20	/	ESPB: Amide local anesthetics + Glucocorticoid	Antiviral therapy	①
Makharita et al. (2015) (11)	Egypt	RCT	70/68	56.8 ± 3.1/ 56.2 ± 3.7	PVB: Amide local anesthetics + Glucocorticoid	Placebo	①, ②
Lin et al. (2021) (10)	China	RCT	26/24	65.2 ± 9.7/ 68.2 ± 9.8	ESPB: Amide local anesthetics	Placebo	①
Abdelwahab et al. (2022) (36)	Egypt	RCT	30/30/30	60.63 ± 7.21/ 59.47 ± 6.69/ 61.3 ± 6.73	PVB: Amide local anesthetics ESPB: Amide local anesthetics	Antiviral therapy	①, ②
Wang et al. (2021) (37)	China	RCT	52/52	62.21 ± 7.15 61.22 ± 11.37	PVB: Amide local anesthetics + Methylene blue	PVB: Amide local anesthetics	①
Patil et al. (2024) (38)	India	RCT	20/20/20	/	ESPB: Amide local anesthetics + Glucocorticoid	Antiviral therapy	①, ②

RCT, randomized controlled trial; PVB, paravertebral block; ESPB, erector spinae plane block; Pulse RF, Pulse radio frequency; ①, Total effective rate; ②, Visual Analogue Scale (VAS).

### 3.3 Risk of bias assessment of included studies

**Figure 2:**
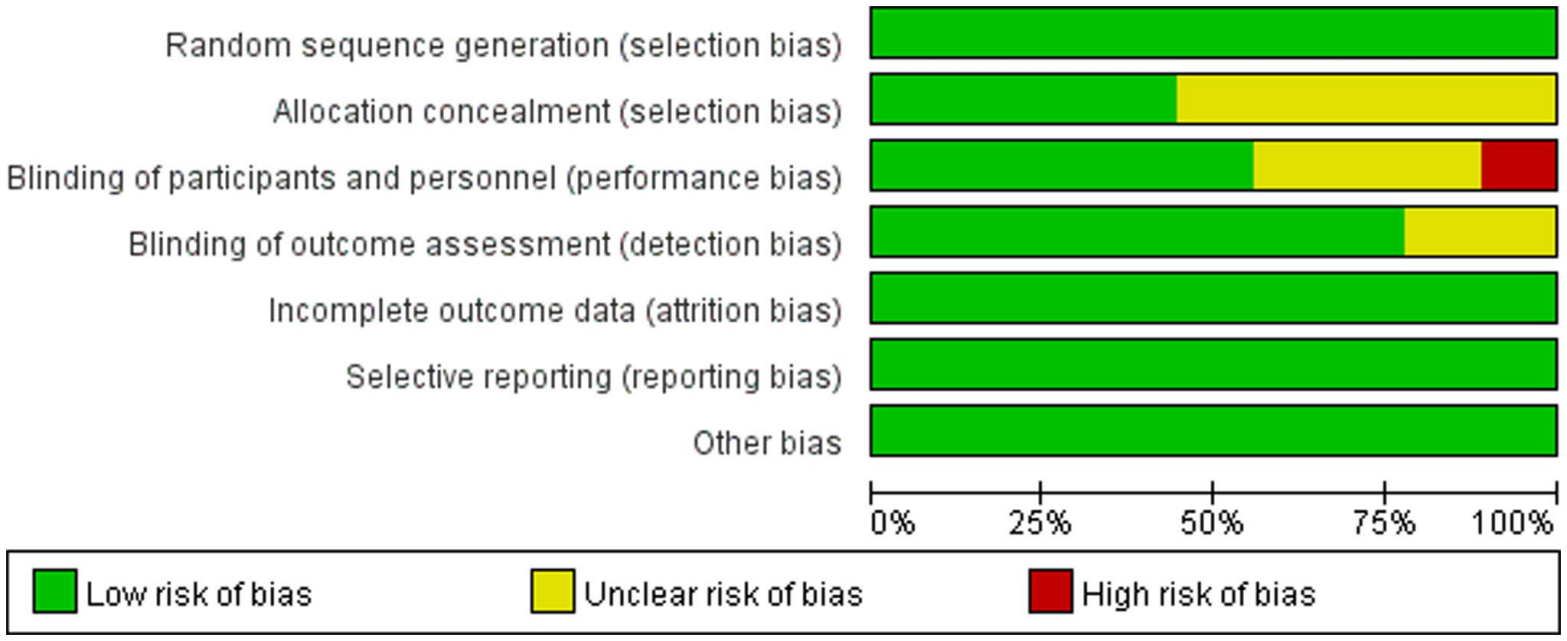
Risk of bias graph.

The risk of bias for the included studies was assessed independently by two reviewers using the Cochrane Handbook for Systematic Reviews of Interventions. For random sequence generation, all 9 studies adopted appropriate randomization methods, such as random number tables and stratified randomization, and were therefore judged as having a low risk of bias. For allocation concealment, 4 studies reported adequate methods and were rated as low risk, while the remaining 5 studies did not provide relevant information and were rated as having an unclear risk. In terms of blinding of participants and personnel, 1 study was rated as high risk, 3 as unclear risk, and 5 as low risk. For blinding of outcome assessment, 2 studies were judged as having an unclear risk, and 7 as low risk. All included studies had complete outcome data and were considered to have a low risk of attrition bias. Other potential sources of bias were not reported in any of the studies and were therefore judged to have a low risk. A summary of the risk of bias assessment is presented in Figure 2.

### 3.4 Network meta-analysis results

#### 3.4.1 SUCRA values and ranking

The SUCRA rankings revealed that, in terms of efficacy for preventing postherpetic neuralgia (PHN), PVB: Amide local anesthetics + Methylene blue > PVB: Methylene blue > ESPB: Amide local anesthetics + Glucocorticoid. Regarding pain relief as measured by the Visual Analogue Scale (VAS), PVB: Amide local a nesthetics + Glucocorticoid > PVB: Amide local anesthetics > ESPB: Amide local anesthetics + Glucocorticoid. Detailed SUCRA values and rankings are provided in Tables 2, 3; Appendixes 2, 3.

**Table 2:** The ordering results of network meta-analysis of total effective rate.

Intervention	Total effective rate	
	SUCRA	Rank
PVB: Amide local anesthetics + Methylene blue	96.0	1
PVB: Methylene blue	64.3	2
ESPB: Amide local anesthetics + Glucocorticoid	64.2	3

**Table 3:** The ordering results of network meta-analysis of Visual Analogue Scale (VAS).

Intervention	VAS	
	SUCRA	Rank
PVB: Amide local a nesthetics + Glucocorticoid	75.0	1
PVB: Amide local anesthetics	67.3	2
ESPB: Amide local anesthetics + Glucocorticoid	57.4	3

#### 3.4.2 Network plot

**Figure 3:**
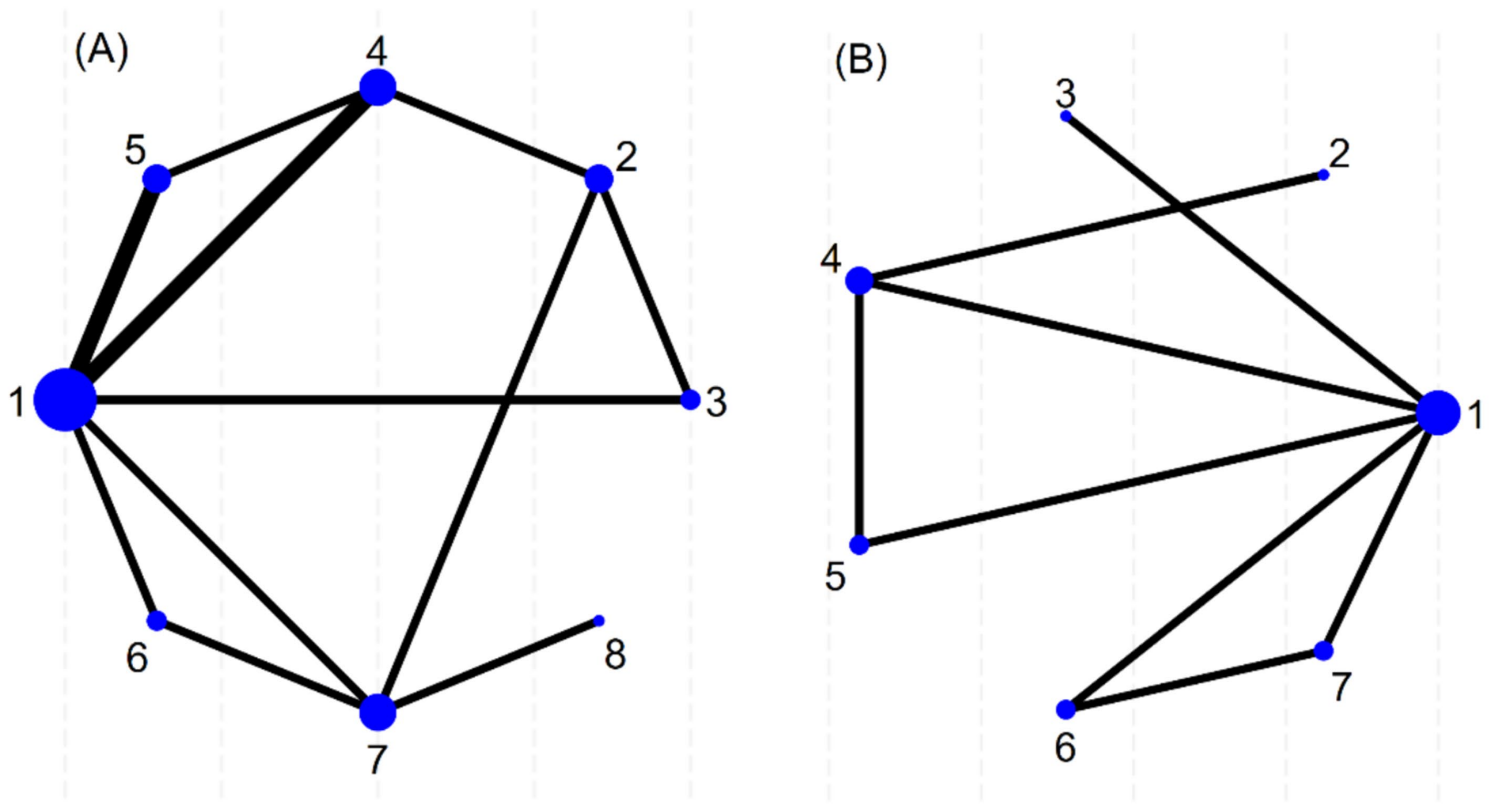
Network figure about efficient evidence. **(A)**: Total effective rate, **(B)**: Visual Analogue Scale, 1: Antiviral therapy, 2: Placebo, 3: PVB: Methylene blue, 4: PVB: Amide local anesthetics + Glucocorticoid, 5: ESPB: Amide local anesthetics + Glucocorticoid, 6: ESPB: Amide local anesthetics, 7: PVB: Amide local anesthetics, 8: PVB: Amide local anesthetics+ Methylene blue.

Prior to data synthesis, the transitivity assumption across the included studies was assessed and found not to be violated. A total of eight different interventions were analyzed and visualized using a network plot (Figure 3). In the plot, each node represents a specific intervention, with the size of the node corresponding to the total sample size for that intervention. The thickness of the lines connecting the nodes indicates the number of studies directly comparing the linked interventions.

#### 3.4.3 Network meta-analysis results for total effective rate

The network meta-analysis of the total effective rate indicated that antiviral therapy was significantly less effective compared to PVB: amide local anesthetics plus methylene blue (MD = 2.63, 95% CI: 1.23 ~ 4.04). A statistically significant difference was also observed between PVB: methylene blue and PVB: amide local anesthetics + methylene blue (MD = 1.19, 95% CI: −0.52 ~ 2.90). Furthermore, PVB: amide local anesthetics + glucocorticoids was significantly different from ESPB: amide local anesthetics (MD = −0.59, 95% CI: −1.81 ~ 0.64), and ESPB: amide local anesthetics + glucocorticoids also differed significantly from ESPB: amide local anesthetics alone (MD = −0.69, 95% CI: −2.15 ~ 0.77). No statistically significant differences were found in the remaining pairwise comparisons (Table 4; Appendix 4).

**Table 4:** Total effective rate network meta-analysis [MD (95% CI)].

Interventions	AT	PB	P: MB	P: ALN + Glu	E: ALN + Glu	E: ALN	P: ALN
AT							
PB	0.41 (−0.52,1.33)						
P: MB	1.44 (0.21,2.67)	1.40 (0.02,2.05)					
P: ALN + Glu	1.33 (0.62,2.04)	0.92 (0.11,1.73)	−0.11 (−1.32,1.10)				
E: ALN + Glu	1.43 (0.40,2.47)	1.03 (−0.22,2.28)	−0.01 (−1.53,1.51)	0.11 (−0.93,1.14)			
E: ALN	0.74 (−0.32,1.80)	0.34 (−0.96,1.64)	−0.70 (−2.25,0.85)	−0.59 (−1.81,0.64)*	−0.69 (−2.15,0.77)*		
P: ALN	1.32 (0.36,2.28)	0.91 (−0.11,1.94)	−0.12 (−1.49, 1.24)	−0.01 (−1.07,1.05)	−0.11 (−1.47,1.24)	0.58 (−0.57,1.72)	
P: ALN + MB	2.63 (1.23,4.04)*	2.23 (0.78,3.68)	1.19 (−0.52,2.90)*	1.31 (−0.17,2.78)	1.20 (−0.50,2.90)	1.89 (0.35,3.43)	1.31 (0.29,2.34)

*Pairwise comparison, *p* < 0.05, P, paravertebral block; E, erector spinae plane block; AT, Antiviral therapy; PB, Placebo; MB, Methylene blue; ALN, Amide local anesthetics; Glu, Glucocorticoid.

#### 3.4.4 Network meta-analysis results for visual analogue scale (VAS)

The network meta-analysis of the Visual Analogue Scale (VAS) scores revealed that, compared with antiviral therapy, several interventions showed statistically significant reductions in pain intensity. These included placebo (MD = −1.86, 95% CI: −3.4 ~ −0.26), PVB: methylene blue (MD = −1.78, 95% CI: −2.85 ~ −0.71), PVB + amide local anesthetics plus glucocorticoids (MD = −2.15, 95% CI: −3.69 ~ −0.60), ESPB: amide local anesthetics plus glucocorticoids (MD = −1.85, 95% CI: −3.52 ~ −0.18), ESPB: amide local anesthetics (MD = −1.43, 95% CI: −2.78 ~ −0.80), and PVB: amide local anesthetics (MD = −1.99, 95% CI: −3.24 ~ −0.74). No statistically significant differences were observed in the remaining pairwise comparisons (Table 5; Appendix 5).

**Table 5:** Visual Analogue Scale network meta-analysis [MD (95% CI)].

Interventions	AT	PB	P: MB	P: ALN + Glu	E: ALN + Glu	E: ALN
AT						
PB	−1.86 (−3.46,-0.26)*					
P: MB	−1.78 (−2.85,-0.71)*	0.08 (−1.85,2.00)				
P: ALN + Glu	−2.15 (−3.69,-0.60)*	−0.29 (−0.71,0.13)	−0.37 (−2.25,1.51)			
E: ALN + Glu	−1.85 (−3.52,-0.18)*	0.01 (−1.82,1.84)	−0.07 (−2.05,1.91)	0.30 (−1.49,2.08)		
E: ALN	−1.43 (−2.78,-0.80)*	0.43 (−1.67,2.52)	0.35 (−1.37,2.07)	0.72 (−1.33,2.77)	0.42 (−1.73,2.56)	
P: ALN	−1.99 (−3.24, 0.74)*	−0.13 (−2.16,1.90)	−0.21 (−1.85,1.43)	0.16 (−1.83,2.14)	−0.14 (−2.22,1.94)	−0.56 (−1.51,0.39)

*Pairwise comparison, *p* < 0.05, P, paravertebral block; E, erector spinae plane block; AT, Antiviral therapy; PB, Placebo; MB, Methylene blue; ALN, Amide local anesthetics; Glu, Glucocorticoid.

### 3.5 Publication bias and sensitivity analysis

**Figure 4:**
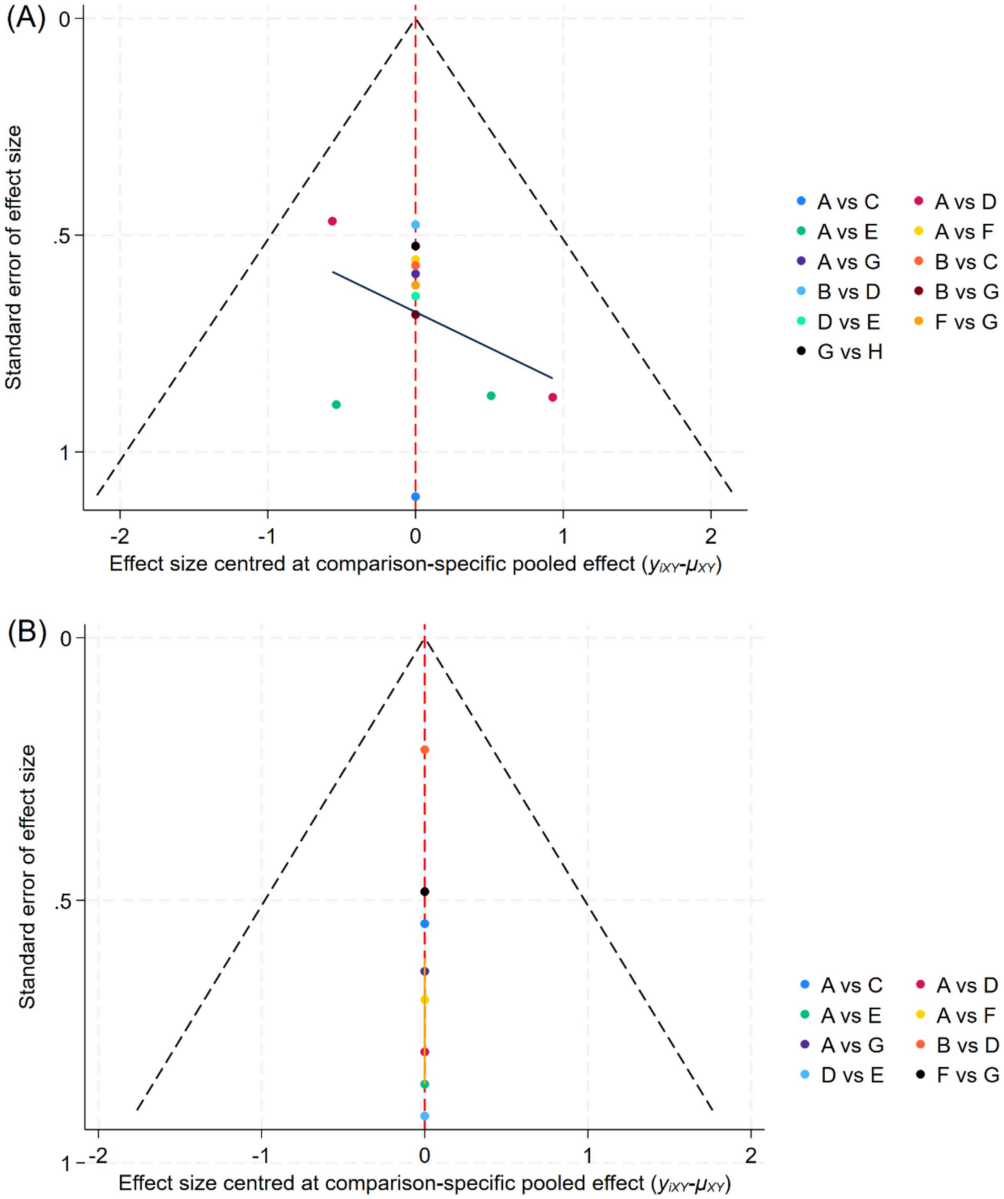
Funnel plot of each outcome indicator. **(A)**: Total effective rate, **(B)**: Visual Analogue Scale, A: Antiviral therapy, B: Placebo, C: PVB: Methylene blue, D: PVB: Amide local anesthetics + Glucocorticoid, E: ESPB: Amide local anesthetics + Glucocorticoid, F: ESPB: Amide local anesthetics, G: PVB: Amide local anesthetics, H: PVB: Amide local anesthetics+ Methylene blue.

The funnel plot indicated a low likelihood of publication bias, suggesting that small sample effects are unlikely to be present (Figure 4). Sensitivity analysis, conducted by sequentially excluding each included study, revealed that no single study had a significant impact on the results, and the conclusions of the study remained unchanged. This suggests that the findings of this analysis are both reliable and stable.

## 4 Discussion

Herpes zoster is a common viral disease that can significantly impact patients’ daily lives and quality of life. Among the affected areas, the chest is a high-incidence region for herpes zoster, and the broad and intense pain it causes has a more severe physical and psychological impact on patients. Some patients may develop postherpetic neuralgia (PHN) after the acute phase, with chronic pain that can persist for months or even years, severely reducing their quality of life. In recent years, interventional treatments have gradually been applied in the prevention and treatment of PHN, offering new strategies to improve patient outcomes. To clarify the relative efficacy of different interventions in the prevention and treatment of PHN in patients with herpes zoster in the chest region, this study conducted a network meta-analysis of eight interventions. The results showed that the top three interventions for improving total effective rate were: PVB: Amide local anesthetics + Methylene blue, PVB: Methylene blue, and ESPB: Amide local anesthetics + Glucocorticoid. The top three interventions for pain relief (VAS scores) were: PVB: Amide local anesthetics + Glucocorticoid, PVB: Amide local anesthetics, and ESPB: Amide local anesthetics + Glucocorticoid. In conclusion, both PVB and ESPB-related interventions demonstrated good efficacy in the prevention and treatment of chest PHN.

### 4.1 Total effective rate

The SUCRA (Surface Under the Cumulative Ranking curve) results provide a quantitative ranking of the comparative efficacy of the included interventions. According to Table 2, PVB: Amide local anesthetics + Methylene blue (SUCRA = 96.0) ranked first, followed by PVB: Methylene blue (SUCRA = 64.3) and ESPB: Amide local anesthetics + Glucocorticoid (SUCRA = 64.2). This ranking suggests that PVB interventions, particularly when combined with methylene blue, have a higher probability of being the most effective in improving the total effective rate of PHN prevention.

From a clinical perspective, these SUCRA results provide important implications for individualized treatment selection. When considering preventive strategies for high-risk thoracic herpes zoster patients (e.g., older adults with severe acute pain), the top-ranked PVB: Amide local anesthetics + Methylene blue should be prioritized due to its robust probability of achieving superior outcomes. The close ranking between PVB: Methylene blue and ESPB: Amide local anesthetics + Glucocorticoid suggests that both options could be considered as second-line strategies when methylene blue or amide anesthetics are contraindicated or unavailable.

PVB, as a relatively simple and effective analgesic technique, allows for the direct injection of anesthetic agents into the paravertebral region at the spinal nerve exit site, achieving high-quality blockade of both the ipsilateral somatic and sympathetic nerves. This results in significant advantages in post-operative analgesia and neuropathic pain management (14, 15). ESPB (erector spinae plane block) is also a simple and safe thoracic pain relief technique, potentially exerting its analgesic effect through its action on the dorsal and ventral branches of the thoracic spinal nerves. Studies have shown that ESPB provides good analgesic effects in the management of chronic neuropathic pain as well as acute post-surgical or post-traumatic pain, demonstrating high clinical application value (16).

Methylene blue (MB), one of the interventions, is an inhibitor of nitric oxide synthase (NOS) and soluble guanylate cyclase (sGC) (17). Research has shown that intravenous infusion of MB significantly reduces pain levels in patients with chronic refractory neuropathic pain. It is a safe and effective analgesic that can be applied to various pain conditions, including intervertebral disc injection (18, 19). The potential mechanism for preventing neuropathic pain involves its anti-inflammatory properties, which can effectively suppress plasma levels of IL-6, TNF-α, and cortisol in PHN patients (20, 21), Additionally, MB has been shown to exert beneficial effects by mitigating mitochondrial dysfunction-induced oxidative stress (22). However, its exact mechanism remains unclear and requires further investigation. Amide local anesthetics are commonly used clinical nerve block anesthetics. However, due to their rapid clearance, the typical duration of nerve blockade or infiltration is relatively short (23, 24). Additionally, all clinically used local anesthetics act on both sensory and motor neurons, leading to the loss of motor function (25). Therefore, the combination of Amide local anesthetics and Methylene blue in PVB provides the most effective intervention for the prevention and treatment of postherpetic neuralgia (PHN) in the chest.

### 4.2 Visual analogue scale

The SUCRA results for pain relief (Table 3) demonstrate that PVB: Amide local anesthetics + Glucocorticoid (SUCRA = 75.0) is the most effective intervention for reducing VAS scores, followed by PVB: Amide local anesthetics (SUCRA = 67.3) and ESPB: Amide local anesthetics + Glucocorticoid (SUCRA = 57.4). These findings indicate that PVB-based interventions remain superior for acute and subacute pain control in thoracic herpes zoster patients. Pairwise comparison analysis further revealed that PVB: Amide local anesthetics + Glucocorticoid has a significantly superior analgesic effect compared to the traditional treatment method, Antiviral therapy, thereby supporting the hypothesis of this study that nerve block therapy could potentially be an effective alternative for preventing PHN in the chest. Traditional local anesthetic treatments have limited effectiveness in preventing PHN, and the analgesics used only provide short-term pain relief for PHN (23, 26). Nerve block therapy addresses this limitation, with studies showing that it allows for the injection of more local anesthetics, providing a longer-lasting analgesic effect (27). Additionally, the use of paravertebral block technology allows local anesthetics to directly permeate the spinal nerves, including the dorsal branch, communicating branches, and sympathetic chain, resulting in a more effective blockade of pain transmission (28). The best intervention in this study not only included the injection of local anesthetics but also incorporated glucocorticoid therapy. Research has shown that glucocorticoids can inhibit the release of inflammatory mediators (such as prostaglandins and cytokines), reduce inflammation around the nerves, and effectively decrease pain sensitivity (29). Furthermore, the addition of glucocorticoids, such as dexamethasone, can extend the analgesic duration of local anesthetics (such as bupivacaine or ropivacaine) by 30–50%, making them especially suitable for chronic pain treatment (30). Glucocorticoids also prolong the duration of action of local anesthetics, reducing the need for repeated injections, thereby minimizing the risk of local anesthetic overdose or excessively high blood drug concentrations, and further enhancing the analgesic effect of the nerve block (31). Therefore, paravertebral nerve block not only increases the local anesthetic dosage and enhances its analgesic effect, but the anti-inflammatory action of glucocorticoids also acts synergistically, making the combination of these two treatments highly effective in preventing the occurrence of chest postherpetic neuralgia.

## 5 Limitations

This study has several methodological limitations that should be considered when interpreting the findings:

(1) The number of included studies and the overall sample size were limited, which may lead to reduced statistical power and increased uncertainty of the pooled estimates.
(2) There was potential clinical and methodological heterogeneity among the included studies, such as differences in patient characteristics, intervention protocols (drug type, dosage, and administration techniques), and outcome measurement methods. Although network meta-analysis can integrate indirect comparisons, such heterogeneity might still influence the reliability of SUCRA-based rankings.
(3) Reporting bias cannot be ruled out. Some included studies lacked detailed reporting of randomization, blinding, or adverse events, which could overestimate the observed treatment effects. Moreover, publication bias may exist since studies with negative or non-significant results are less likely to be published.
(4) The imbalance in the number of comparisons and sample sizes between interventions might distort the relative ranking probabilities, particularly for less-studied treatment strategies.
(5) The SUCRA scores, while informative for ranking interventions, do not account for differences in study quality or risk of bias and should be interpreted as statistical probabilities rather than definitive clinical hierarchies.

Given these limitations, the results of this network meta-analysis should be interpreted with caution, and high-quality, large-scale randomized controlled trials are needed to validate these findings.

## 6 Conclusion

In conclusion, the results of this study indicate that PVB is superior to ESPB in both reducing VAS scores and improving overall effectiveness. Specifically, PVB: Amide local anesthetics + Methylene blue and PVB: Amide local anesthetics + Glucocorticoid are the most effective interventions for the two outcome measures in this study. These findings suggest that PVB may become an effective treatment option for preventing postherpetic neuralgia (PHN) in the future, and the combination of local anesthetics with other effective medications may offer even better therapeutic outcomes.

## Data Availability

The datasets generated and analyzed during the current study, as well as the analysis code used for the network meta-analysis, are available from the corresponding author upon reasonable request.
