# Correction: Comparative effectiveness of nerve block strategies for preventing postherpetic neuralgia in thoracic herpes zoster: a network meta-analysis

**DOI:** 10.3389/fneur.2025.1697557

**Published:** 2025-09-15

**Authors:** Wensheng Lu, Shengze He, Qi Liu, Yaozu Gu, Jie Bai

**Affiliations:** ^1^Department of Anesthesiology, Lanzhou University Second Hospital, Lanzhou, China; ^2^The Second Clinical Medical School, Lanzhou University, Lanzhou, China

**Keywords:** herpes zoster, nerve block, acute zoster-related pain, postherpetic neuralgia, erector spinae plane block, paravertebral block, network meta-analysis

In the published article, the original funding statement was corrected from: “This work was supported by the Natural Science Foundation of Gansu Province, China (Grant No. 22JR5RA920)” to: “This work was supported by the Natural Science Foundation of Gansu Province of China (23JRRA0980), Cuiying Scientific Training Program for Undergraduates of the Second Hospital & Clinical Medical School, Lanzhou University (CYXZ2024-22).”

The original version of this article has been updated.

