# Supplementary material for: Comparative effectiveness of nerve block strategies for preventing postherpetic neuralgia in thoracic herpes zoster: a network meta-analysis
Source: Front Neurol. 2025 Aug 18;16:1612871. doi: 10.3389/fneur.2025.1612871 (PMC12399379; doi:10.3389/fneur.2025.1612871)
Supplement: Supplementary file 1 [file Table_1.docx]

**Appendix 1**

Medline retrieval strategy

| Search number | Query | Results |
| --- | --- | --- |
| 1 | ((((((((("Anesthesia, Epidural"[Mesh]) OR "Anesthesia, Local"[Mesh]) OR "Nerve Block"[Mesh]) OR "Stellate Ganglion"[Mesh]) OR "Superior Cervical Ganglion"[Mesh]) OR "Anesthetics, Local"[Mesh]) OR "Injections, Spinal"[Mesh]) OR "Ganglia, Spinal"[Mesh]) OR "Intercostal Nerves"[Mesh]) NOT "Blood Patch, Epidural"[Mesh] | 123,256 |
| 2 | (((((((((((((((Epidural Anesthesia[Title/Abstract]) OR (Local Anesthesia[Title/Abstract])) OR (Nerve Block[Title/Abstract])) OR (Nerve Blocks[Title/Abstract])) OR (Nerve Blockade[Title/Abstract])) OR (Nerve Blockades[Title/Abstract])) OR (Stellate Ganglion[Title/Abstract])) OR (Stellate Ganglia[Title/Abstract])) OR (Stellate Ganglias[Title/Abstract])) OR (Superior Cervical Ganglia[Title/Abstract])) OR (Epidural Injections[Title/Abstract])) OR (Epidural Injection[Title/Abstract])) OR (Spinal Injections[Title/Abstract])) OR (Spinal Injection[Title/Abstract])) OR (intercostal nerve[Title/Abstract])) OR (dorsal root ganglia[Title/Abstract]) | 60,549 |
| 3 | #1 OR #2 | 123,257 |
| 4 | "Neuralgia, Postherpetic"[Mesh] | 1,529 |
| 5 | ((((((PHN[Title/Abstract]) OR (postherpetic neuralgia[Title/Abstract])) OR (post herpetic neuralgia[Title/Abstract])) OR (post-herpetic neuralgia[Title/Abstract])) OR (postherpetic pain[Title/Abstract])) OR (post herpetic pain[Title/Abstract])) OR (post-herpetic pain[Title/Abstract]) | 4,888 |
| 6 | #4 OR #5 | 5,102 |
| 7 | ("Neuralgia"[Mesh]) OR "Pain"[Mesh] | 487,855 |
| 8 | (neuralgia[Title/Abstract]) OR (pain[Title/Abstract]) | 873,272 |
| 9 | #7 OR #8 | 1,037,325 |
| 10 | "Herpes Zoster"[Mesh] | 13,899 |
| 11 | (((zoster[Title/Abstract]) OR (shingles[Title/Abstract])) OR (zona[Title/Abstract])) OR (VZV[Title/Abstract]) | 32,033 |
| 12 | #10 OR #11 | 40,634 |
| 13 | #9 AND #12 | 4,597 |
| 14 | #6 OR #13 | 7,254 |
| 15 | "Randomized Controlled Trials as Topic"[Mesh] | 184,636 |
| 16 | ((((((randomized controlled trials[Title/Abstract]) OR (randomized controlled[Title/Abstract])) OR (case–control[Title/Abstract])) OR (clinical trial[Title/Abstract])) OR (intervention[Title/Abstract])) OR (controlled trial[Title/Abstract])) OR (randomized controlled study[Title/Abstract]) | 1,513,637 |
| 17 | #15 OR #16 | 1,610,317 |
| 18 | #3 AND #14 AND #17 | 74 |

**Appendix 3** Total effective rate cumulative probability graph


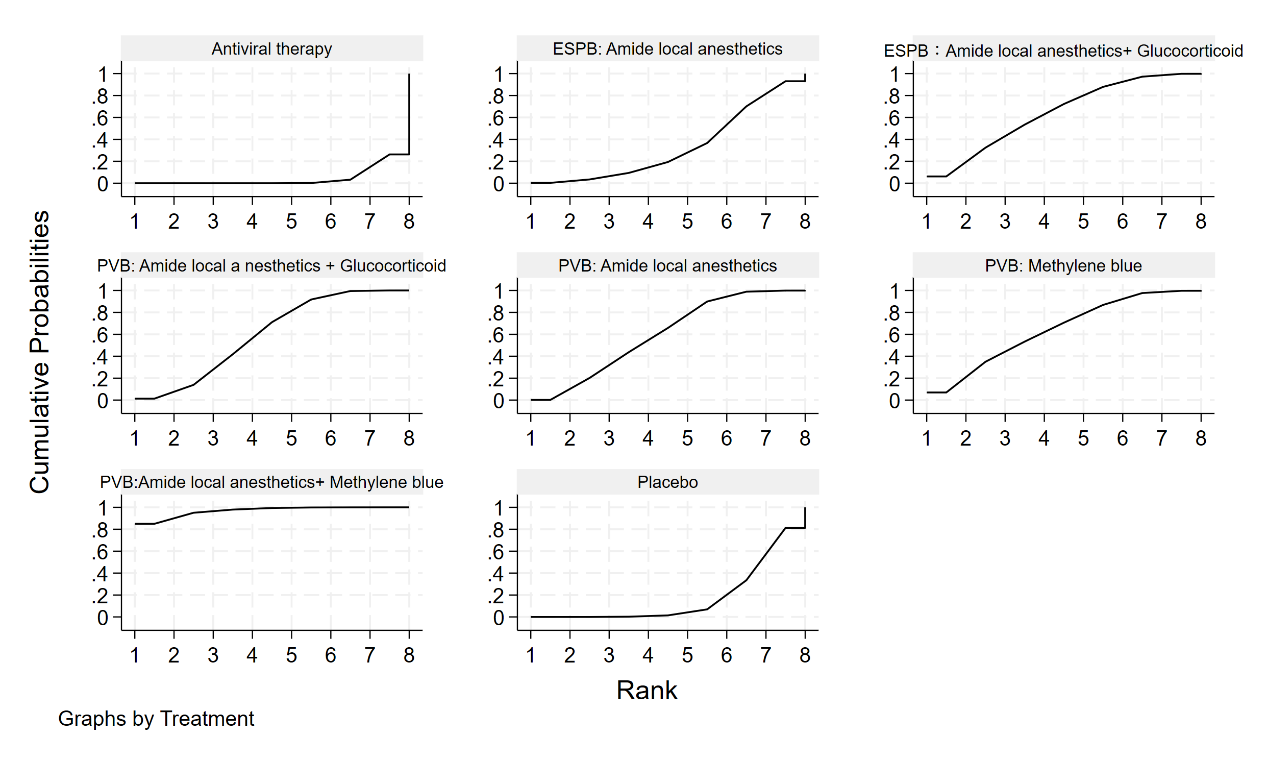


**Appendix 3** Visual Analogue Scale cumulative probability graph


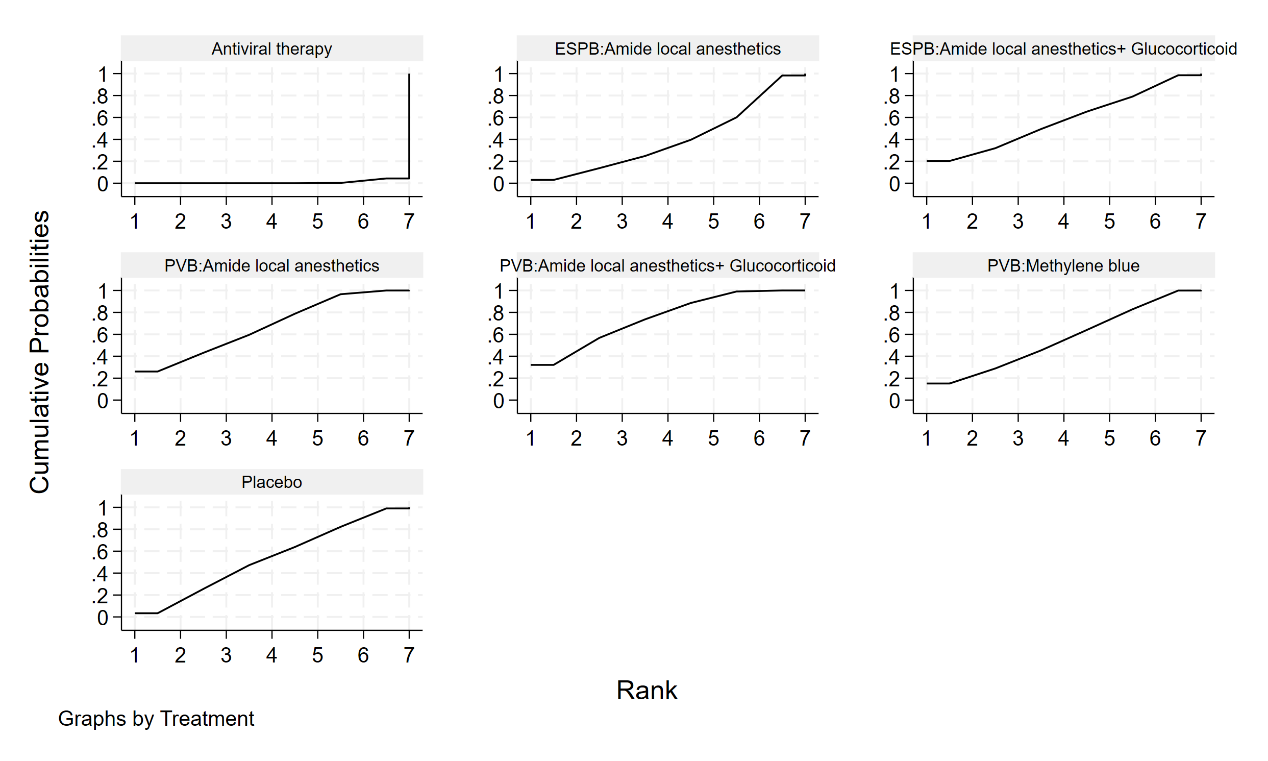


PVB: paravertebral block, ESPB: erector spinae plane block

**Appendix 4** Total effective rate network Meta forest map


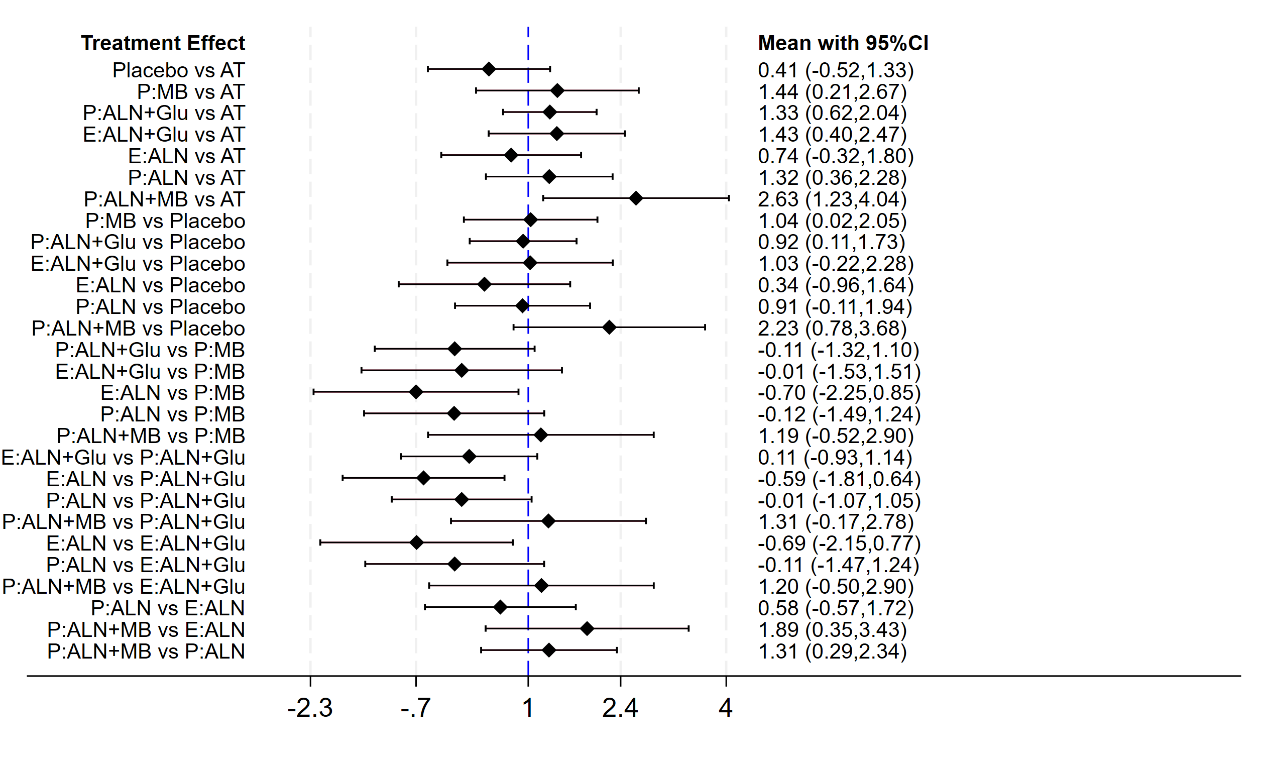


P: paravertebral block, E: erector spinae plane block, AT: Antiviral therapy, PB Placebo, MB: Methylene blue, ALN: Amide local anesthetics, Glu: Glucocorticoid

**Appendix 5** Visual Analogue Scale network Meta forest map


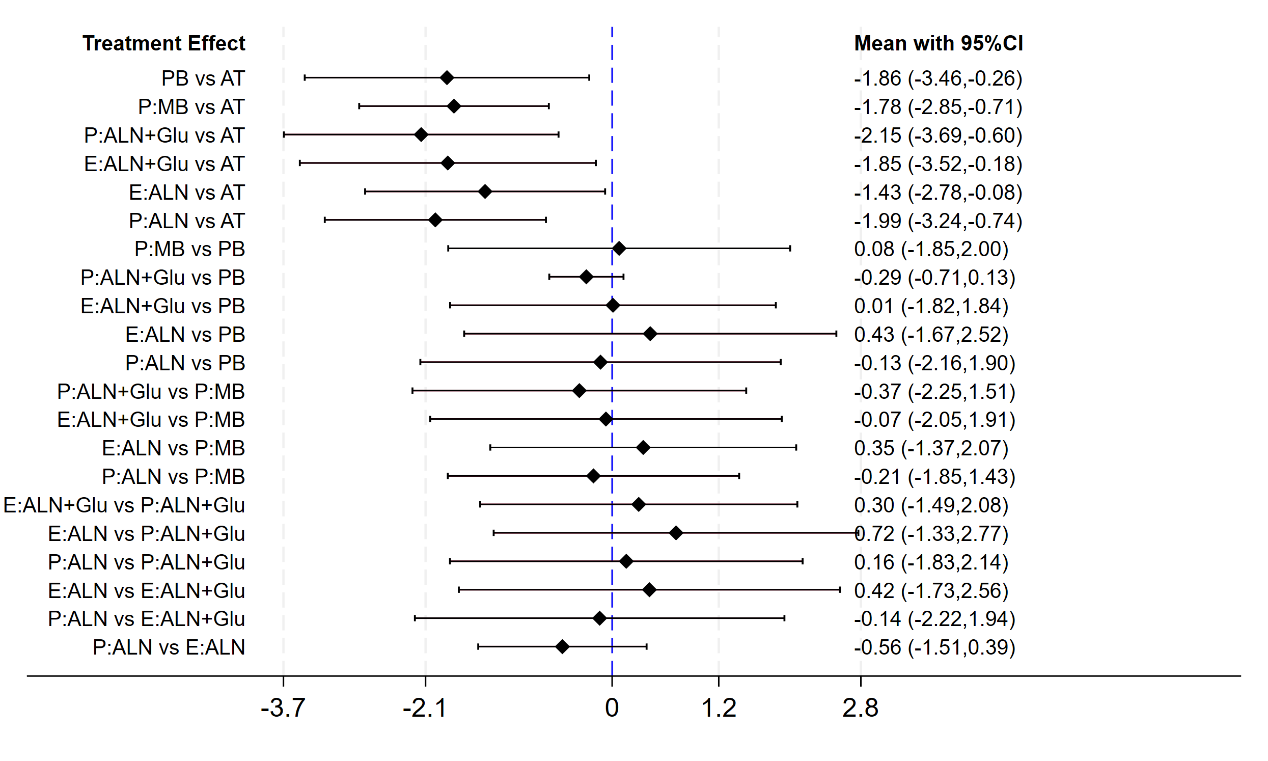


P: paravertebral block, E: erector spinae plane block, AT: Antiviral therapy, PB Placebo, MB: Methylene blue, ALN: Amide local anesthetics, Glu: Glucocorticoid
